# Handwashing knowledge, attitudes, and practices during the COVID-19 pandemic in Saudi Arabia: A non-representative cross-sectional study

**DOI:** 10.1038/s41598-021-96393-6

**Published:** 2021-08-18

**Authors:** Osama Al-Wutayd, Ali E. Mansour, Ahmad Hamad Aldosary, Hamdan Z. Hamdan, Manal A. Al-Batanony

**Affiliations:** 1 Department of Family and Community Medicine, Unaizah College of Medicine and Medical Sciences, Qassim University, Unaizah, Saudi Arabia; 2 Department of Public Health and Community Medicine, Damietta Faculty of Medicine, Al Azhar University, Cairo, Egypt; 3 Department of Basic Medical Sciences, Unaizah College of Medicine and Medical Sciences, Qassim University, Unaizah, Saudi Arabia; 4 Department of Biochemistry, Faculty of Medicine, Al-Neelain University, Khartoum, Sudan; 5 Department of Community Medicine and Public Health, Menofia Faculty of Medicine, Menofia University, Al Minufiyah, Egypt

**Keywords:** Infection, Risk factors

## Abstract

Handwashing (HW) with water and soap is one of the cheapest and most effective ways of protecting oneself and others against the coronavirus. Here, the HW knowledge, attitudes, and practices of Saudi adults were assessed during the COVID-19 pandemic using a cross-sectional study conducted between May 8 and June 8, 2020, during a partial lockdown period. A web-based validated questionnaire was distributed through different social media platforms, and the sociodemographic characteristics of the participants, seven items related to knowledge, four items related to attitudes, and thirteen items related to the practice of HW were assessed. A total of 1323 (51% male and 49% female) adults from all regions of Saudi Arabia responded to the questionnaire. The overall mean (± *SD*) was 5.13 (± 1.18) for knowledge of HW and COVID-19, 2.79 (± 0.77) for attitude toward HW, and 7.8 (± 2.56) for HW practice. A multiple linear regression analysis revealed factors associated with knowledge to be age and family income. Sex, educational level, family income, and HW knowledge were associated with negative and neutral attitude, whereas age, sex, family income, and HW knowledge were associated with practice. These results suggest that HW knowledge was strongly associated with positive attitudes toward HW and correct HW practice in Saudi adults during the COVID-19 lockdown.

## Introduction

On December 31, 2019, the World Health Organization (WHO) Regional Office in Wuhan City, Hubei Province, China, reported cases of pneumonia of an unknown cause. Most patients worked at or lived around the local Huanan seafood wholesale market, where live animals were also on sale^1^. On January 7, 2020, a novel coronavirus was identified by the Chinese Center for Disease Control and Prevention (CDC) from the throat-swab sample of a patient^2^. The symptoms ranged from those of a common cold to those of more severe diseases, such as Middle East respiratory syndrome (MERS-CoV) and severe acute respiratory syndrome (SARS-CoV), which are examples of a large family of viruses called coronaviruses (CoV)^2,3^. Symptoms of the new virus, named 2019-nCoV, may appear 2–14 days after exposure in the form of a fever, cough, and shortness of breath. Most patients have mild symptoms and a good prognosis, but some develop severe pneumonia, pulmonary edema, acute respiratory distress syndrome, or multiple organ failure, and some have died^4^. 2019-nCoV, like other respiratory viruses, spreads by droplet infection and commonly enters the body via the eyes, nose, or throat. Hand contact is also a commonly reported way of spreading the virus from human to human^5^. Recent studies indicate that people 60 years or older are more vulnerable to the severe form of the disease compared to children, who might show milder symptoms, or may even be asymptomatic^6^. As of September 15, 2020 (3:57 PM CET), the case fatality rate of the coronavirus disease caused by 2019-nCoV (COVID‐19) was reported to be approximately 3.1% (926,544/29,155,581), compared to the 9.6% (774/8096) reported for the SARS‐CoV epidemic^7^ and 34.4% (858/2494) in the MERS‐CoV outbreak from 2012 to the present day^8^. In Saudi Arabia, the first confirmed case was on March 23, 2020 in Qatif, the eastern region of the kingdom, and the case was a traveler returning from Iran, a neighboring country^9^. Thereafter, the cases started to escalate and reached a peak on June 18, 2020, where the daily reported number of cases was 4919, with 39 deaths^10^. The WHO^11^ and Saudi Ministry of Health^12^ advised the global population to take many precautions to reduce the chances of being infected by or spreading COVID-19, beginning with handwashing (HW). Regular and thorough HW with water and soap for at least 40 s and hand rub for 20 s using a sanitizer with a minimum alcohol concentration of 60%—both of which kill viruses on the hands—are the easiest, cheapest, most effective, and most important methods for preventing the spread of disease, especially during a global pandemic.

The role of community knowledge in dealing with pandemics is well appreciated^13,14^. Studies documenting the level of knowledge, attitude, and practice (KAP) toward COVID-19 and hand washing in Saudi Arabia, especially during the lockdown period, are scant^15–18^. One study found that level of education is associated with the KAP of COVID-19^19^. We hypothesize that high educational level and high family income are associated with the level of KAP toward hand washing. Therefore, this study sought to assess the HW knowledge, attitudes, and practices among Saudis during the COVID-19 pandemic. This study is important in addressing the gaps in the KAP literature on hand washing at the community level. These findings can be used to tailor a health educational program designed by health authorities to promote positive attitudes and effective handwashing practices.

## Materials and methods

### Study setting and design

This was a web-based cross-sectional non-representative study conducted among Saudi nationals in the Kingdom of Saudi Arabia between May 8 and June 8, 2020, during the COVID-19 pandemic lockdown.

### Sample size calculation

The sample size was calculated using an open epi-calculator^20^ based on the practice of handwashing frequency, guided by a study during the outbreak of the SARS epidemic in Hong Kong^21^. Assuming adequate power (80%), and a type I error of 5%, the sample size was determined by considering that the practice frequency of > 10 time HW per day among males was 40% and 50% among females. The minimum sample size was 1060, taking into account 30% of expected missing and incomplete responses. We approached 1738 subjects and obtained 1323 complete responses, a response rate of 76.1%.

### Questionnaire preparation and description

A self-administered questionnaire written in Arabic language (S1) was developed by the investigators using information published recently to address the objectives of the study^12,15,22^. A pilot study was conducted with 25 males and 25 females of different ages, who were asked to complete the questionnaire and then report on whether it was easy to understand and what the estimated time for completion was. These results are not included in this report, but some modifications were made to ensure clarity and ease of understanding the questions. The face and content validities of the questionnaire were reviewed by three experts (two epidemiologists and one consultant in infection control; all had more than 10 years of experience in their fields). Each expert was approached individually by the principal investigator to review the questionnaire and add their comments. The experts performed the reviewing task separately and made notes and responses, with an average of two times before final approval.

The questionnaire scoring system is described as follows: first, in the knowledge and practice sections, 1 point was assigned for each correct response/active practice and zero for incorrect response/passive practice. Second, in the attitude section, 1 point was assigned for positive attitude, while zero was assigned for negative and neutral attitude.

Cronbach’s alpha was calculated for each section: knowledge (0.64), attitude (0.63), and practice (0.62) of the questionnaire. The final version of the questionnaire comprised 34 multiple-choice questions in four sections: sociodemographic data (10 items), knowledge of HW (7 items), attitudes regarding HW (4 items), and HW practices (13 items).

### Data collection

The web-based questionnaire, which included forced-fields questions, was placed on Google-Forms platform. Then distributed via several social media platforms. Its title, the objective of the study, the voluntary nature of participation, declarations of confidentiality and anonymity, estimated duration (5–6 min), and URL link were included on the cover page. The inclusion criteria were Saudi adults aged 18 years or older living in the Kingdom of Saudi Arabia. The questionnaire was randomly shared on social media and personally shared with the investigators’ Saudi national contacts lists. The frontpage of the questionnaire showed the study title, purposes, and inclusion criteria (Saudi National and ≥ 18 years) to ensure respondent eligibility. The study procedure was approved by the institutional review board at Qassim University, and conducted in accordance with the ethical principles for medical research involving human subjects as described in the Declaration of Helsinki. Informed consent was obtained from all participants in this study. This study’s ethical approval was obtained from the Qassim Region Research Ethics Committee on May 5, 2020 (Reference No. 19-11-02).

### Statistical analysis

The data were received in Excel sheet and manually surveyed to exclude duplicated responses, after that exported to STATA version 16 for statistical analysis. The data are presented as a mean (*SD*) for continuous variables or as a number (percentage) for categorical variables. Comparisons between continuous variables were performed using either the Student’s *t*-test or one-way ANOVA for more than two groups, and the chi-squared test was used to compare the categorical variables. A multivariable regression analysis was performed to identify the determinants of HW knowledge, attitudes, and practices. Variables such as the respondent’s age category, sex, educational level, marital status, the presence of elderly people in the same household, and chronic disease were considered independent variables; knowledge, attitudes, and practices were the dependent variables. Variables with p < 0.25 in the bivariate analysis were included in the multivariable analysis. A multiple linear regression analysis was performed to determine the factors that affected the knowledge score. A multiple logistic regression analysis was performed to determine the factors that affected attitudes and practices. Odds ratios (ORs), beta coefficients, and 95% confidence intervals (CIs) are reported, as appropriate. A *p* value of less than 0.05 was considered strong evidence against the null hypothesis.

## Results

### Descriptive characteristics of the participants

A total of 1,323 participants were included in the study: 356 (27%) participants were aged 30–39 years, 678 (51%) were males, 805 (61%) had a bachelor’s degree, 1,060 (80%) were married, 544 (41%) had a family income from 2666 US Dollars (USD) to 5333 USD, 333 (25%) had chronic illnesses, and 390 (29%) had an elderly person living in the same house. The descriptive characteristics are detailed in Table 1.

**Table 1:** Descriptive characteristics and comparison of the mean HW knowledge scores.

Characteristics	Category	N (%)	Knowledge score (mean ± *SD*)	*p*
Age group	18–29 years	227 (17)	5.21 ± 1.17	< 0.001^a^*
	30–39 years	356 (27)	5.33 ± 1.10	
	40–49 years	309 (23)	5.17 ± 1.19	
	50–59 years	258 (20)	4.96 ± 1.15	
	≥ 60 years	173 (13)	4.82 ± 1.25	
Gender	Male	678 (51)	5.06 ± 1.23	0.032 ^b^*
	Female	645 (49)	5.20 ± 1.11	
Educational level	≤ Secondary school	362 (27)	4.98 ± 1.12	0.004^a^*
	Bachelor’s degree	805 (61)	5.17 ± 1.19	
	Postgraduate	156 (12)	5.31 ± 1.20	
Marital status	Married	1060 (80)	5.15 ± 1.18	0.565^a^
	Single	208 (16)	5.05 ± 1.19	
	Other (widowed, divorced)	55 (4)	5.13 ± 0.98	
Family income (USD)	< 2666	506 (38)	5.02 ± 1.24	< 0.001^a^*
	2666–5333	544 (41)	5.11 ± 1.13	
	≥ 5333	273 (21)	5.38 ± 1.11	
History of chronic conditions	Yes	333 (25)	4.91 ± 1.24	< 0.001^b^*
	No	990 (75)	5.21 ± 1.14	
Elderly living with you in the same house	Yes	390 (29)	5.11 ± 1.20	0.587 ^b^
	No	933 (71)	5.14 ± 1.17	

^a^ANOVA, ^b^Independent *t*-test, *p < 0.05.

### Knowledge of handwashing

HW-related knowledge was assessed by seven items, which are provided in Table 2. The average knowledge score was 5.13 (*SD* = 1.18, range 1–7), and the rate of correct responses ranged from 30 to 94%. Based on the bivariate analysis, older age groups and male participants had significantly lower knowledge scores (*p* < 0.001 and *p* = 0.032, respectively), whereas those with higher education levels, higher family incomes, and free from chronic illnesses had significantly higher knowledge scores (*p* = 0.004, *p* ≤ 0.001, and *p* < 0.001, respectively) (Table 1). The multiple linear regression showed that those in the age group (50–59 vs. 18–29) (− 0.29 [− 0.51, − 0.08]; 0.008), age group (60 + vs. 18–29) (− 0.43 [− 0.68, − 0.18]; 0.001) and family income (5333 USD + vs. < 2666 USD) (0.42 [0.24, 0.60]; < 0.001] positively influenced the knowledge mean score, as shown in Table 3.

**Table 2:** Questionnaire on handwashing knowledge (K), attitudes (A), and practices (P) during the COVID-19 pandemic in Saudi Arabia (n = 1323).

Variable	Responses	Determination/score	N (%)
K1: What is the best HW method to prevent coronavirus?	HW with water only/hand sanitizer	Incorrect/0	73( 6)
	HW with soap and water	Correct/1	1250 (94)
K2: What is the minimum time period for HW with soap and water to prevent coronavirus?	5 s/10 s/I don’t know	Incorrect/0	348 (26)
	40 s	Correct/1	975 (74)
K3: What is the lowest alcohol concentration in hand sanitizer that prevents coronavirus?	30%/40%/50%/I don’t know	Incorrect/0	921 (70)
	60%	Correct/1	402 (30)
K4: Is using warm water necessary and important during HW to prevent coronavirus?	Yes/I don’t know	Incorrect/0	556 (42)
	No	Correct/1	767 (58)
K5/6: What are the modes of transmission of coronavirus?	K5: touch-contaminated surfaces (yes)	Correct/1	1061 (80)
	K6: droplets during sneezing, talking (yes)	Correct/1	1150 (87)
K7: Have you seen a video explaining the proper method of HW in the last 3 months?	Yes	Correct/1	1185 (90)
	No	Incorrect/0	138 (10)
A1: Do you think that you are vulnerable to infection with coronavirus?	Yes	Positive/1	559 (42)
	Maybe/No	Negative/0	764 (58)
A2: Do you think that HW with soap and water reduces the possibility of coronavirus infection?	Yes	Positive/1	1230 (93)
	Maybe/No	Negative/0	93 (7)
A3: Do you think that, while wearing gloves, you should not touch your face?	Yes	Positive/1	1267 (96)
	Maybe/no	Negative/0	56 (4)
A4: Do you hesitate to direct your family members to wash their hands with soap and water when needed, such as when returning from public places?	No	Positive/1	625 (47)
	Maybe/yes	Negative/0	698 (53)
**Technique of HW with soap and water**			
P1: Do you wash the insides and the backs of your hands?	Always	Correct/1	1148 (87)
	Sometimes/never	Incorrect/0	175 (13)
P2: Do you wash between your fingers?	Always	Correct/1	1062 (80)
	Sometimes/never	Incorrect/0	261 (20)
P3: Do you wash your wrists?	Always	Correct/1	836 (63)
	Sometimes/never	Incorrect/0	487 (37)
P4: Do you wash your fingertips?	Always	Correct/1	1097 (83)
	Sometimes/never	Incorrect/0	226 (17)
P5: Do you wash your thumbs?	Always	Correct/1	1049 (79)
	Sometimes/never	Incorrect/0	274 (21)
P6: Do you wash under your nails?	Always	Correct/1	639 (48)
	Sometimes/never	Incorrect/0	684 (52)
P7: Do you dry your hands with a clean towel after washing them?	Always	Correct/1	838 (63)
	Sometimes/never	Incorrect/0	785 (37)
**Duration of the entire procedure**			
P8: How long do you wash your hands with soap and water?	Less than 40 s/I don’t know	Incorrect/0	686 (52)
	40–60 s	Correct/1	637 (48)
**Frequency of HW per day**			
P9: How many times do you wash your hands each day with soap and water?			
	Ten times or less	Incorrect/0	962 (73)
	More than ten times	Correct/1	361 (27)
**Key times to wash hands**			
P10–13: At which times do you wash your hands with soap and water?	P10: after visiting public places (yes)	Correct/1	1249 (94)
	P11: after touching any high-touch surfaces outside the house (yes)	Correct/1	1077 (81)
	P12: after removing gloves (yes)	Correct/1	1004 (76)
	P13: after removing a mask (yes)	Correct/1	854 (65)

**Table 3:** Multiple linear regression on factors associated with handwashing knowledge.

Variable	Coefficient	95% CI	*p* value
Age group (50–59 vs. 18–29)	− 0.29	− 0.51, − 0.08	0.008*
Age group (60 + vs. 18–29)	− 0.43	− 0.68, − 0.18	0.001*
Education level (postgraduate vs. bachelor’s degree)	0.17	− 0.03, 0.38	0.093
Family income in USD (5333 + vs. < 2666)	0.42	0.24, 0.60	< 0.001*
Family income in USD (2666–5333 vs. < 2666)	0.13	− 0.01, 0.28	0.076
Chronic illness (yes vs. no)	− 0.13	− 0.29, 0.02	0.098

Other variables (age group 30–39 vs. 18–29 years, 40–49 vs. 18–29 years; gender; ≤ secondary school vs. bachelor’s degree) were not mentioned in the table (p > 0.1).

*p < 0.05.

### Attitudes toward handwashing

HW-related attitudes were assessed by four items, which are presented together with their associated responses in Table 2. The average attitude score was 2.79 (*SD* = 0.77, range 0–4), and the positive attitude rates ranged from 42 to 96%. The bivariate analysis showed strong evidence of associations between attitudes and several independent variables, as shown in Table 4. The multiple logistic regression found negative and neutral responses to A1—Do you think that you are vulnerable to infection with coronavirus?—more often among females (vs. males, aOR 1.79, *p* < 0.001), among those with less than a secondary school education (vs. a bachelor’s degree, aOR 1.45, *p* = 0.008), and less often among a family income of + 5333 USD (vs. < 2666 USD, aOR 0.64, *p* = 0.007), a postgraduate degree (vs. a bachelor’s degree, aOR 0.68, *p* = 0.041), and among those with HW knowledge scores (aOR 0.82, *p* < 0.001). Negative and neutral responses to A2—Do you think that HW reduces the possibility of coronavirus infection?—were less common among those with HW knowledge scores (aOR 0.67, *p* < 0.001), and among a family income of 2666 USD—5333 USD (vs. < 2666 USD, aOR 0.49, *p* = 0.006)*.* Negative and neutral responses to A3—Do you think that, while wearing gloves, you should not touch your face?—were less common in those with HW knowledge scores (aOR 0.71, *p* = 0.002) and with female vs. male (aOR 0.53, *p* = 0.040). Negative and neutral responses to A4—Do you hesitate to direct your family members to wash their hands when needed, such as when returning from public places?—were more common among those with ≤ secondary school education (vs. a bachelor’s degree, aOR 1.77, *p* ≤ 0.001) and among those who have an elder person living with them (aOR 1.31, *p* < 0.047). Such responses were less common with HW knowledge scores (aOR 0.72, *p* < 0.001), those with family incomes between + 5333 USD (vs. < 2666 USD, aOR 0.61, *p* = 0.003), those who were single vs. married (aOR 0.65, p = 0.050), and those with postgraduate educational level (vs. bachelor’s degree, aOR 0.57, p = 0.003) (Table 5).

**Table 4:** Attitudes to handwashing based on sociodemographic variables.

Characteristics	A1	A2	A3	A4
	n (%)	n (%)	n (%)	n (%)
**Age group, years**	Positive	Positive	Positive	Positive
18–29	84 (37)	212 (93)	219 (96)	120 (53)
30–39	161 (45)	334 (94)	346 (97)	174 (49)
40–49	131 (42)	287 (93)	297 (96)	132 (43)
50–59	100 (39)	238 (92)	249 (97)	129 (50)
≥ 60	83 (48)	159 (92)	156 (90)	70 (40)
Chi**-**squared	0.113	0.915	0.003*	0.044*
**Sex**				
Female	228 (35)	595 (92)	628 (97)	316 (49)
Male	331 (49)	635 (94)	639 (94)	309 (46)
Chi**-**squared	< 0.001*	0.316	0.005*	0.203
**Education level**				
≤ Secondary school	118 (33)	326 (90)	342 (94)	124 (34)
Bachelor’s degree	351 (44)	757 (94)	773 (96)	401 (50)
Postgraduate	90 (58)	147 (94)	152 (97)	100 (64)
Chi-squared	< 0.001*	0.039*	0.260	< 0.001*
**Marital status**				
Single	79 (38)	194 (93)	197 (95)	111 (53)
Married	462 (44)	985 (93)	1016 (96)	486 (46)
Other	18 (33)	51 (93)	54 (98)	28 (55)
Chi**-**squared	0.112	0.982	0.501	0.119
**Family income (USD)**				
< 2666	190 (38)	455 (90)	486 (96)	205 (41)
2666–5333	225 (41)	518 (95)	522 (96)	259 (48)
+ 5333	144 (53)	257 (94)	259 (95)	161 (59)
Chi**-**squared	< 0.001*	0.002*	0.710	< 0.001*
**Chronic illnesses**				
Yes	148 (44)	307 (92)	315 (95)	155 (47)
No	411 (42)	923 (93)	952 (96)	470 (47)
Chi**-**squared	0.349	0.521	0.219	0.769
**Elderly living with you in the same house**				
Yes	177 (45)	363 (93)	368 (94)	174 (45)
No	382 (41)	867 (93)	899 (97)	451 (48)
Chi**-**squared	0.136	0.922	0.100	0.216
**Knowledge score**				
Mean [SD]	5.3 [1.2]	5.2 [1.2]	5.2 [1.2]	5.4 [1.1]
*t*-test	< 0.001*	< 0.001*	< 0.001*	< 0.001*

Knowledge score (negative attitude, mean [*SD*] for A1 = 5.01 [1.2], A2 = 4.5 [1.3], A3 = 4.6 [1.3], A4 = 4.9 [1.2]).

*p < 0.05.

**Table 5:** Multiple logistic regression on factors associated with negative attitudes.

Variable	aOR (95% CI)	*p* value
**A1: Negative and neutral (vs positive)**		
Sex (female vs. male)	1.79 (1.42, 2.27)	< 0.001*
Family income in USD (+ 5333 vs. < 2666)	0.64 (0.46, 0.89)	0.007*
Education level (≤ secondary school vs. bachelor’s degree)	1.45 (1.10,1.91)	0.008*
Education level (postgraduate vs. bachelor’s degree)	0.68 (0.47, 0.98)	0.041*
HW knowledge score	0.82 (0.75, 0.91)	< 0.001*
**A2: Negative and neutral (vs. positive)**		
Education level (≤ secondary school vs. bachelor’s degree)	1.49 (0.93, 2.37)	0.093
Family income in USD (2666–5333 vs. < 2666)	0.49 (0.30, 0.82)	0.006*
HW knowledge score	0.67 (0.56, 0.79)	< 0.001*
**A3: Negative and neutral (vs. positive)**		
Age group (60 + vs. 18–29 years)	2.41 (0.96, 6.07)	0.061
Sex (female vs. male)	0.53 (0.29, 0.97)	0.040*
HW knowledge score	0.71 (0.57, 0.88)	0.002*
**A4: Negative and neutral (vs. positive)**		
Education level (≤ secondary school vs. bachelor’s degree)	1.77 (1.34, 2.32)	< 0.001*
Education level (postgraduate vs. bachelor’s degree)	0.57 (0.39, 0.83)	0.003*
Marital status (never married vs. married)	0.65 (0.42, 0.99)	0.050*
Family income in USD (+ 5333 vs. < 2666)	0.61 (0.44, 0.85)	0.003*
Does an elderly person live with you? (yes vs. no)	1.31 (1.00, 1.71)	0.047*
HW knowledge score	0.72 (0.65, 0.79)	< 0.001*

*p < 0.05.

### Handwashing practice

Bivariate analysis revealed that factors significantly associated with never/sometimes follow of HW techniques were male sex (*p* = 0.031), family income + 5.333 USD (*p* = 0.008), and mean of HW knowledge score of 5.08 (*p* = 0.010). Factors associated with washing the hand for less than 40 s were healthy persons versus those with chronic disease (*P* = 0.009) and a mean HW knowledge score of 4.92 (*p* ≤ 0.001). Factors that were associated with the frequency of HW < 10 times/day were age group (18–29) (*p* = 0.005), male sex (*p* ≤ 0.001) and mean HW knowledge score 5.07 (*p* = 0.002).

#### Bivariate analysis

Factors that were associated with not washing hands after visiting public places were age group ≥ 60 years (*p* = 0.002), educational level ≤ secondary school (*p* ≤ 0.001), and mean HW knowledge score 4.18 (*p* ≤ 0.001). Not washing one’s hand after touching a high touch surface outside was significantly associated with an age group ≥ 60 years (*p* ≤ 0.001), male sex (*p* = 0.001), educational level ≤ secondary school (*p* ≤ 0.008) and mean HW knowledge score of 4.47 (*p* ≤ 0.001). Not washing hands after removing gloves was associated with an age group ≥ 60 years (*p* = 0.001), male sex (*p* ≤ 0.001), educational level ≤ secondary school (*p* ≤ 0.003), mean HW knowledge score of 4.48 (*p* ≤ 0.001), and mean HW attitude score of 2.71 (*p* = 0.033). Not washing hands after removing masks was significantly associated with an age group ≥ 60 years (*p* = 0.017), male sex (*p* = 0.032), educational level ≤ secondary school (*p* ≤ 0.029) and mean HW knowledge score of 4.47 (*p* ≤ 0.001). Table 6.

**Table 6:** Bivariate analysis of factors associated with handwashing practice (technique, duration, and frequency).

Characteristics	n	Follow HW technique		Duration of HW		Frequency of HW/day		
	1323	Never/sometimes n (%) 929 (70)	Always n (%) 394 (30)	< 40 s n (%) 686 (52)	40–60 s n (%) 637 (48)	≤ 10 times n (%) 962 (73)	> 10 times n (%) 361 (27)	
**Age group, years**								
18–29	227	174 (77)	53 (23)	125 (55)	102 (45)	179 (79)	48 (21)	
30–39	356	238 (67)	118 (33)	188 (53)	168 (47)	261 (73)	95 (27)	
40–49	309	213 (69)	96 (31)	148 (48)	161 (52)	203 (66)	106 (34)	
50–59	258	181 (70)	77 (30)	140 (54)	118 (46)	184 (71)	74 (29)	
≥ 60	173	123 (71)	50 (29)	85 (49)	88 (51)	135 (78)	38 (22)	
Chi-square		0.151		0.390		0.005*		
**Sex**								
Female	645	435 (67)	210 (33)	317 (49)	328 (51)	427 (66)	218 (34)	
Male	678	494 (73)	184 (27)	369 (54)	309 (46)	535 (79)	143 (21)	
Chi-square		0.031*		0.055		< 0.001*		
**Education level**								
≤ Secondary school	362	245 (68)	117 (32)	170 (47)	192 (53)	262 (72)	100 (28)	
Bachelor’s degree	805	573 (71)	232 (29)	438 (54)	367 (46)	586 (73)	219 (27)	
Postgraduate	156	111 (71)	45 (29)	78 (50)	78 (50)	114 (73)	42 (27)	
Chi-square		0.464		0.055		0.983		
**Marital status**								
Never married	208	153 (74)	55 (26)	106 (51)	102 (49)	163 (78)	45 (22)	
Married	1060	740 (70)	320 (30)	552 (52)	508 (48)	764 (72)	296 (28)	
Other	55	36 (65)	19 (35)	28 (51)	27 (49)	35 (64)	20 (36)	
Chi-square		0.409		0.948		0.054		
**Family income (USD)**								
< 2666	506	334 (66)	172 (34)	251 (50)	255 (50)	375 (74)	131 (26)	
2666–5333	544	386 (71)	158 (29)	292 (54)	252 (46)	394 (72)	150 (28)	
+ 5333	273	209 (77)	64 (23)	143 (52)	130 (48)	193 (71)	80 (29)	
Chi-square		0.008*		0.411		0.583		
**Chronic illnesses**								
Yes	333	231 (69)	102 (31)	152 (46)	181 (54)	236 (71)	97 (29)	
No	990	698 (71)	292 (29)	534 (54)	456 (46)	726 (73)	264 (27)	
Chi-square		0.695		0.009*		0.383		
**Elderly living in the same house**								
Yes	390	271 (69)	119 (31)	198 (51)	192 (49)	284 (73)	106 (27)	
No	933	658 (71)	275 (29)	488 (52)	445 (48)	678 (73)	255 (27)	
Chi-square		0.707		0.610		0.955		
**Knowledge score**								
Mean [*SD*]		5.08 [1.2]	5.26 [1.1]	4.92 [1.2]	5.4 [1.1]	5.07 [1.2]	5.29 [1.1]	
*t*-test		0.010*		< 0.001*		0.002*		
**Attitude score**								
Mean [*SD*]		2.78 [0.78]	2.81[0.74]	2.80 [0.8]	2.7 [0.7]	2.80 [0.8]	2.75 [0.8]	
*t*-test		0.417		0.467		0.312		

Characteristics	Washing hands after visiting public places		Washing hands after touching a high-touch surface outside		Washing hands after removing gloves		Washing hands after removing mask	
	No n (%) 74 (6)	Yes n (%) 1249 (94)	No n (%) 246 (19)	Yes n (%) 1077 (81)	No n (%) 319 (24)	Yes n (%) 1004 (76)	No n (%) 469 (35)	Yes n (%) 854 (65)
**Age group, years**								
18–29	7 (3)	220 (97)	30 (13)	197 (87)	57 (25)	170 (75)	79 (35)	148 (65)
30–39	9 (3)	347 (97)	47 (13)	309 (87)	66 (19)	290 (81)	103 (29)	253 (71)
40–49	25 (8)	284 (92)	66 (21)	243 (79)	69 (22)	240 (78)	113 (37)	196 (63)
50–59	18 (7)	240 (93)	60 (23)	198 (77)	65 (25)	193 (75)	100 (39)	158 (61)
≥ 60	15 (9)	158 (91)	43 (25)	130 (75)	62 (36)	111 (64)	74 (43)	99 (57)
Chi-square	0.002*		< 0.001*		0.001*		0.017*	
**Sex**								
Female	32 (5)	613 (95)	96 (15)	549 (85)	126 (20)	519 (80)	210 (33)	435 (67)
Male	42 (6)	636 (94)	150 (22)	528 (78)	193 (28)	485 (72)	259 (38)	419 (62)
Chi-square	0.329		0.001*		< 0.001*		0.032*	
**Education level**								
≤ Secondary school	35 (10)	327 (90)	84 (23)	278 (77)	109 (30)	253 (70)	145 (40)	217 (60)
Bachelor’s degree	37 (5)	768 (95)	143 (18)	662 (82)	182 (23)	623 (77)	280 (35)	525 (65)
Postgraduate	2 (1)	154 (99)	19 (12)	137 (88)	28 (18)	128 (82)	44 (28)	112 (72)
Chi-square	< 0.001*		0.008*		0.003*		0.029*	
**Marital status**								
Never married	11 (5)	197 (95)	30 (14)	178 (86)	53 (25)	155 (75)	64 (31)	144 (69)
Married	58 (5)	1002 (95)	208 (20)	852 (80)	259 (24)	801 (76)	387 (37)	673 (63)
Other	5 (9)	50 (91)	8 (15)	47 (85)	7 (13)	48 (87)	18 (33)	37 (67)
Chi-square	0.512		0.155		0.124		0.261	
**Family income (USDs)**								
< 2666	29 (6)	477 (94)	94 (19)	412 (81)	130 (26)	376 (74)	172 (34)	334 (66)
2666–5333	26 (5)	518 (95)	103 (19)	441 (81)	123 (23)	421 (77)	204 (38)	340 (62)
+ 5333	19 (7)	254 (93)	49 (18)	224 (82)	66 (24)	207 (76)	93 (34)	180 (66)
Chi-square	0.435		0.943		0.506		0.428	
**Chronic illnesses**								
Yes	23 (7)	310 (93)	56 (17)	277 (83)	79 (24)	254 (76)	113 (34)	220 (66)
No	51 (5)	939 (95)	190 (19)	800 (81)	240 (24)	750 (76)	356 (36)	634 (64)
Chi-square	0.228		0.335		0.848		0.504	
**Elderly living in the same house**								
Yes	27 (7)	363 (93)	75 (19)	315 (81)	105 (27)	285 (73)	138 (35)	252 (65)
No	47 (5)	886 (95)	171 (18)	762 (82)	214 (23)	719 (77)	331 (35)	602 (65)
Chi-square	0.174		0.700		0.122		0.974	
**Knowledge score**								
Mean [*SD*]	4.18 [1.11]	5.18 [1.15]	4.47 [1.2]	5.28 [1.1]	4.48 [1.2]	5.34 [1.1]	4.71 [1.2]	5.36 [1.1]
*t*-test	< 0.001*		< 0.001*		< 0.001*		< 0.001*	
**Attitude score**								
Mean [*SD*]	2.74 [0.92]	2.79 [0.75]	2.80 [0.9]	2.78 [0.7]	2.71 [0.9]	2.81 [0.7]	2.79 [0.8]	2.78 [0.7]
*t*-test	0.603		0.709		0.033*		0.750	

#### Following the correct handwashing technique

Using the correct HW technique was assessed by seven items, which are listed together with their scores in Table 2. The correct HW technique was always observed by 394 (30%) participants.

Multiple logistic regression analysis (Table 7) showed that never or sometimes following the correct HW technique was more common among males (vs. females, aOR 1.31, *p* = 0.031), those with family incomes above + 5333 USD (vs. < 2666 USD, aOR 1.92, *p* ≤ 0.001) and those with family incomes from 2666 USD to < 5333 USD (vs. < 2666 USD, aOR 1.37, *p* = 0.022), those with HW knowledge score (aOR 0.85, *p* = 0.003) and among those aged 18–29 years (vs. all other age groups).

**Table 7:** Multiple logistic regression of factors associated with handwashing practice (technique, duration, frequency, and key time).

Variable	aOR (95%CI)	*p* value
**P1–7: Never/sometimes perform HW with soap and water (vs. always)**		
Age group (30–39 vs. 18–29)	0.61 (0.42–0.90)	0.013*
Age group (40–49 vs. 18–29)	0.61 (0.41–0.91)	0.015*
Age group (50–59 vs. 18–29)	0.59 (0.39–0.90)	0.013*
Age group (60 + vs. 18–29)	0.56 (0.35–0.89)	0.015*
Sex (male vs. female)	1.31 (1.02–1.67)	0.031*
Family income in USD (+ 5,333 vs. < 2,666)	1.92 (1.35–2.73)	< 0.001*
Family income in USD (2,666 to < 5,666 vs. < 2,666)	1.37 (1.05–1.79)	0.022*
HW knowledge score	0.85 (0.77–0.95)	0.003*
**P8: Less than the recommended HW duration (vs. 40 s +)**		
Sex (male vs. female)	1.27 (1.01–1.59)	0.038*
Education level (≤ secondary vs. bachelor’s degree)	0.72 (0.56–0.93)	0.012*
Chronic illness (yes vs. no)	0.63 (0.49–0.82)	0.001*
HW knowledge score	0.70 (0.64–0.78)	< 0.001*
**P9: HW less than ten times a day with soap and water (vs. > 10 times)**		
Age group (40–49 vs. 18–29)	0.48 (0.29–0.79)	0.004*
Age group (50–59 vs. 18–29)	0.61 (0.36–0.79)	0.063
Sex (male vs. female)	1.91 (1.47–2.46)	< 0.001*
HW knowledge score	0.85 (0.76–0.95)	0.004*
**P10: Not washing hands after visiting public places**		
Age group (40–49 vs. 18–29)	2.95 (1.22–7.15)	0.017*
Age group (50–59 vs. 18–29)	2.36 (0.93–6)	0.072
Age group (60 + vs. 18–29)	2.72 (1.02–7.24)	0.046*
Education level (≤ secondary vs. bachelor’s degree)	2.04 (1.23–3.37)	0.005*
Education level (postgraduate vs. bachelor’s degree)	0.27 (0.06–1.14)	0.075
HW knowledge score	0.53 (0.43–0.65)	< 0.001*
**P11: Not washing hands after touching a high-touch surface outside**		
Sex (male vs. female)	1.47 (1.08–2)	0.013*
Education level (postgraduate vs. bachelor’s degree)	0.60 (0.35–1.03)	0.065
HW knowledge score	0.58 (0.51–0.65)	< 0.001*
**P12: Not washing hands after removing gloves**		
Sex (male vs. female)	1.45 (1.09–1.92)	0.009*
Education level (≤ secondary school vs. bachelor’s degree)	1.41 (1.04–1.90)	0.026*
Marital status (other vs. married)	0.45 (0.19–1.05)	0.065
HW knowledge score	0.54 (0.48–0.61)	< 0.001*
**P13: Not washing hands after removing mask**		
HW knowledge score	0.63 (0.56–0.69)	< 0.001*

Follow HW technique (never/sometimes vs. always); duration of HW (< 40 s vs. 40 s +); frequency of HW (≤ 10 times vs. > 10 times).

*p < 0.05.

#### Following the correct handwashing duration

A multiple logistic regression analysis showed that only male sex was a risk factor for washing hands for less than 40 s (aOR 1.27, *p* = 0.038), while other factors, such as educational level (less than secondary vs. a bachelor’s degree), chronic illness (yes vs. no), and handwashing knowledge scores showed protection against washing hands for less than the recommended duration, as shown in Table 7.

#### Daily handwashing frequency

Among the factors investigated, only male sex (aOR 1.91, *p* < 0.001) indicated a risk for washing one’s hands less than ten times daily, whereas age (40–49 years vs. 18–29 years) and age group (50–59 years vs. 18–29 years) and handwashing high knowledge score predicted HW more than ten times daily, as shown in Table 7.

#### Not washing hands and after visiting public places

Educational level (≤ secondary vs. a bachelor’s degree aOR 2.04, *p* = 0.005), age group 40–49 years vs. 18–29 years (aOR 2.95, *p* = 0.017), age group (0–59 years vs. 18–29 years (aOR 2.36, *p* = 0.072), and age group 60 + years vs. 18–29 years (aOR 2.72, *p* = 0.046) were associated with not washing hands after visiting public places. However, postgraduate educational level and high knowledge score were protected.

#### Not washing hands after touching a high-touch surface outside

Among the investigated factors, only males were associated with not washing hands after touching surfaces (aOR 1.47, *p* = 0.013). High knowledge score handwashing showed protection against not washing hands after touching a high touch surface.

#### Not washing hands after removing gloves

Male sex (aOR 1.45, *p* = 0.009) and educational level ≤ secondary school vs. bachelor’s degree (aOR 1.41, *p* = 0.026) were associated with increased risk of not washing hands after removing gloves. High hand washing knowledge score showed a protective effect.

#### Not washing hands after removing a face mask

Among the investigated factors, only the hand-washing knowledge score (aOR 0.63, *p* = 0.001) was significantly associated with washing hands after removing face mask.

## Discussion

Previous studies have investigated HW as a preventive measure against many infectious respiratory system diseases, such as SARS, H1N1 influenza, and avian influenza^23–25^, but most of these studies were conducted on healthcare staff^26–28^. The current study was community-based and involved respondents from all regions of the Kingdom of Saudi Arabia.

The major finding of this study was that the overall mean knowledge level in this sample was 5.13. This indicates that the majority of the study population had good knowledge of HW to prevent COVID-19 infection. This finding is in agreement with that of Mahdi et al*.*^17^ who conducted a study about hand hygiene knowledge, attitude, and practice (KAP) among domestic Hajj pilgrims and in contrast to Mahdi et al*.*^18^ who conducted a similar KAP study among visitors to the Prophet’s Mosque in Al Madinah, which reported a moderate level of knowledge. Yet, this comparison should be considered with caution, as both mentioned studies investigated both Saudi and non-Saudi subjects. It is worth mentioning that during the COVID-19 pandemic, the health authorities in Saudi Arabia launched a broad, multi-language health education campaign that targeted all populations, including both Saudi and non-Saudi nationals. This campaign included national TV programs, the Ministry of Health website, social media platforms, mobile phone SMS messages, and direct public awareness sessions. The broadcast materials contained information about the virus, its transmission, and precautionary measures, including HW^29^.

The WHO recommends HW—along with many other measures—for reducing the transmission rate of COVID-19 infections worldwide^30^. Alcohol-based hand rubbing solution is one of the methods recommended by the WHO for hand cleansing, and it has determined the effective alcohol concentration to be a minimum of 60% and ideally 80%^1,31^. In this study, only 30% of the respondents correctly identified the effective alcohol concentration of alcohol-based hand sanitizers. The level of knowledge reported in this study was associated with many variables in the bivariate analysis, including age, educational level, gender, family income, and the medical history of the respondents. It was noted that those aged 30–39 and those with high educational levels had significantly higher mean knowledge scores than those aged over 60 or those with average educational levels. Moreover, women scored significantly better than men—in the multivariable analysis. A high monthly family income was significantly associated with a higher mean knowledge score, which is in line with a similar survey about SARS in Hong Kong^32^. High income likely allows individuals to obtain smart phones, smart TVs, and secure internet access, ensuring access to more methods of health education and awareness that are broadcast in the media. Those with low incomes may have limited resources for such access. Old age decreased the knowledge score compared to younger participants. This may be because younger participants are more expert and familiar with using smartphones and the internet and are more active in social media than older participants, and most of the health education programs and campaigns are easily accessible through these technological innovations^33^.

In this study, the attitude level was typically aligned with the level of knowledge, as the overall attitude score was 70%. Less than half of the respondents (42%) thought that they were vulnerable to COVID-19. This finding is in contrast to a study from Hong Kong, in which 84.8% felt they or a member of their family would contract SARS^34^. This low vulnerability prediction is attributed to the fact that at the time of data collection, the number of cases globally was around 4 million cases, and in Saudi Arabia only 44,000 cases in 14th May 2020^35^. It is worth mentioning that male respondents felt that they had a greater risk of contracting COVID-19 than females did. This is in contrast to a study in Hong Kong, which reported that females felt they were more likely to contract Swine flu than males^36^. This may be because women were more likely to stay at home because their children were banned from visiting public places during the partial lockdown. In this study, female respondents had more appropriate attitudes toward touching their faces while wearing gloves and washing their hands after removing their gloves. This attitude reflects the psychology of females toward dealing with the risk of infection^32^, and it may help women feel less vulnerable to COVID-19 infection, as was observed in this study.

Another finding is that high educational levels increase the feeling of being vulnerable to contracting COVID-19, not touching your face while wearing gloves, and actively directing family members to wash their hands when needed. This may be explained by more education being correlated with a greater awareness of the seriousness of this disease and an increased sense of vulnerability. However, increased education was also associated with positive attitudes, as most of the moderately educated and highly educated respondents believed that HW reduces infection risk, which is consistent with a recent study from Saudi Arabia by Al-Hanawi^15^. Notably, educational level, high income, and high knowledge were linked in the present study.

Another important finding in this study is that only 47% of the participants thought that they could actively ask their family members to cleanse their hands after outdoor activities; those family members might prefer staying home rather than going outside, or they may follow precautionary measures without the need for extra instructions. In the bivariate analysis, many factors were predictive of attitude, including age, sex, educational level, and family income. More specifically, an elderly age group (60 + years) was more likely than other age groups to think that touching one’s face while wearing gloves is allowed. This negative attitude is not surprising, as this age group had the lowest knowledge score.

This study found that 71.8% of respondents always followed the best HW technique, but only 48% washed their hands for at least 40 s. 27% wash their hands more than ten times daily. This result is far lower than the 43% reported by Lau et al. for Hong Kong, although the respondents in that study performed HW more than ten times only when traveling across mainland China^37^. Interestingly, 94% of the respondents washed their hands after visiting public places, which is far higher than the 63.6% reported from China at the time of the SARS outbreak^38^. Furthermore, 81% of our respondents washed their hands after touching a high-touch surface, consistent with the 81.2% reported for Hong Kong participants but higher than the 48.3% reported for Singaporeans at the time of SARS^39^. In this study, younger respondents, male respondents, and those with low HW knowledge scores had the lowest HW frequency. This remained significant in the multivariable analysis. Women had better knowledge than men, reflecting that women practice HW more accurately and more frequently than men, which is in line with three recent studies from Saudi Arabia that reported women as more likely to adhere to self-protective measures than men^15,16,39^. Women are known to have a lower threshold for uncleanliness than men, and they may therefore be more concerned about infections and cleansing knowledge and behavior^40^. In the multivariable analysis, a larger monthly income was negatively associated with the accurate practice of HW, contrary to a study from the Philippines^41^. Perhaps, those with high family income have a job environment that does not necessitate frequent hand washing compared to the other jobs. The presence of chronic illnesses did not affect the technique or frequency of washing hands, but it affected the duration of HW, as the majority of those with chronic illness washed their hands for more than 40 s. This remained significant in the multivariable analysis. Washing one’s hands for 40–60 s is recommended by the WHO^42^. It is important to understand that it is not only water and soap that cleanse the hands, but also mechanical forces and the techniques of HW. To correctly perform the techniques requires at least 40–60 s^42^.

In the literature, older persons living in the house may encourage other family members to practice HW frequently, and other family members may practice more HW out of caution and concern about transmitting the infection to an older person^43^. In this study, the presence of elderly people living in the home did not affect HW practices either positively or negatively. There is no clear explanation for this. When interpreting the results of this study, there are some limitations that should be considered. First, this study was conducted during a period of social distancing and was a self-administered questionnaire conducted online. The distribution of the questionnaire depended on the researchers’ connections; it was therefore a convenience sample rather than a random one. Thus, selection bias, report bias, and cohort differences cannot be ruled out. Second, although non-Saudi represents around 37% of the total Saudi Arabia populations^21^, the study population was limited to the Saudi nationals only. Therefore, this study is non-representative. Third, responses to some of the questions in the questionnaire might be easily predicted, which may affect the reliability of the results. Fourth, yes/no/may-be types of questions were used over using a Likert scale. This approach is powerful in addressing the respondent’s knowledge, yet it may underestimate the respondent’s attitude and practice views. Fifth, the anxiety levels of the respondents were not measured, which may have explained more of the findings. Sixth, the study design was cross-sectional, so causality cannot be determined. Therefore, further studies using different designs with revered/distracted questionnaire answers are needed.

## Supplementary Information

Supplementary Information.
