# Supplementary material for: Handwashing knowledge, attitudes, and practices during the COVID-19 pandemic in Saudi Arabia: A non-representative cross-sectional study
Source: Sci Rep. 2021 Aug 18;11:16769. doi: 10.1038/s41598-021-96393-6 (PMC8373984; doi:10.1038/s41598-021-96393-6)
Supplement: Supplementary file 1 — Supplementary Information. [file 41598_2021_96393_MOESM1_ESM.docx]

***The following questions include the socio-demographic characteristics of the participant:***

1. **Age:** (1) 18-29 years (2) 30-39 years

( 3) 40-49 years ( 4) 50-59 years ( 5) ≥ 60 years.

1. **Sex:** ( 1) Female ( 2) Male.
2. **Education level:** (1 ) ≤ secondary school (2 ) bachelor's degree

( 3) postgraduate

1. **Marital Status:** (1) Married ( 2) Single (3 ) Widowed (4 ) Divorced
2. **The average family monthly income** (USD):

(1) <2,666 (2) 2,666–5,333 (3) ≥5,333

1. **Do you have any chronic disease (such as diabetes, hypertension, bronchial asthma, etc.(.?**

(1) Yes (2) No

1. **Does an elderly (older than 60 years) live with you at home?**

(1) Yes (2) No

***The following questions are about knowledge of hand hygiene:***

1. **What is the best HW method to prevent coronavirus??**

( ) Washing hands with water only.

( ) Washing hands with soap and water.

( ) Using alcohol-based hand rub (hand sanitizer).

1. **What is the minimum time period for HW with soap and water to prevent coronavirus?**

( 1) 5 seconds ( 2) 10 seconds ( 3) 40 seconds (4 ) I don't know

1. **What is the lowest alcohol concentration in alcohol-based hand rubs (hand sanitizer) to prevent Corona virus?**

(1) 30% (2) 40% (3) 50% (4) 60% (5) I don’t know

**4- Is using warm water necessary and important during HW to prevent coronavirus?**

(1) Yes (2) No (3) I don't know

**5/6- What are the modes of transmission of Coruna virus?**

(5) By touch-contaminated surfaces [yes, No]

(6) By droplets during sneezing, coughing, talking [yes, No]

1. **Have you seen a video explaining the proper method of HW in the last 3 months?**

                        (1) Yes (2) No

***The following questions are about attitude regarding hand hygiene:***

1. **Do you think that you are vulnerable to infection with Coronavirus?**

(1) Yes (2) No (3) Maybe

1. **Do you think that HW with soap and water reduces the possibility of coronavirus infection?**

(1) Yes (2) No (3) Maybe

1. **Do you think that while wearing gloves, you should not touch your face?**

(1) Yes (2) No (3) Maybe

**4- Do you hesitate to direct your family members to wash their hands with soap and water when needed, such as when returning from public places?**

(1) Yes (2) No (3) Maybe

***The following questions about practicing hand hygiene:***

1. **Do you wash the inside and the back of your hands?**

(1 ) Always (2 ) sometimes ( 3) Never

1. **Do you wash between your fingers?**

)1) Always (2 ) sometimes ( 3) Never

1. **Do you wash your wrists?**

)1) Always (2 ) sometimes ( 3) Never

1. **Do you wash fingertips?**

)1) Always (2 ) sometimes ( 3) Never

1. **Do you wash your thumb?**

)1) Always (2 ) sometimes ( 3) Never

1. **Do you wash under your nails?**

)1) Always (2 ) sometimes ( 3) Never

1. **Do you dry your hands with a clean towel after washing them?**

)1) Always (2 ) sometimes ( 3) Never

1. **How long do you wash your hands with soap and water?**

(1) Less than 40 seconds ( 2) 40-60 seconds ( 3) I don't know

1. **How many times do you wash your hands each day with soap and water?** (1 ) ten times or less (2) more than ten times

**10/13At which time do you wash your hands with soap and water?**

( ) After visiting public places such as groceries (yes, No).

 ( ) After touching any high-touch surfaces outside the house (yes, No).

 ( )After removing gloves (yes, No).

) ) After removing a mask (yes, No).
